# Diagnostic and Prognostic Values of MANF Expression in Hepatocellular Carcinoma

**DOI:** 10.1155/2020/1936385

**Published:** 2020-04-22

**Authors:** Jingyi He, Guangbing Li, Xihan Liu, Liye Ma, Peng Zhang, Jiayao Zhang, Shunzhen Zheng, Jianping Wang, Jun Liu

**Affiliations:** ^1^Department of Hepatobiliary Surgery and Center of Organ Transplantation, Shandong Provincial Hospital Affiliated to Shandong University, Jinan 250021, Shandong Province, China; ^2^School of Medicine, Shandong University, Jinan 250021, Shandong Province, China

## Abstract

Hepatocellular carcinoma (HCC) is one of the most common malignant tumors, and its prognosis is still poor. Mesencephalic astrocyte-derived neurotrophic factor (MANF) plays a key role in endoplasmic reticulum stress. ER stress plays a key role in HCC carcinogenesis. To confirm the clinical and prognostic value of MANF in HCC, we investigated the expression level of MANF in HCC as recorded in databases, and the results were verified by experiment. Survival analysis was probed by the Kaplan–Meier method. Cox regression models were used to ascertain the prognostic value of MANF in HCC tissue microarray. The diagnostic value of MANF in HCC was evaluated by receiver operating characteristic curve analysis. Potential correlation between MANF and selected genes was also analyzed. Results showed that MANF was overexpressed in HCC. Patients with high MANF expression levels had a worse prognosis and higher risk of tumor recurrence. Furthermore, the expression level of MANF had good diagnostic power. Correlation analysis revealed potential regulatory networks of MANF in HCC, laying a foundation for further study of the role of MANF in tumorigenesis. In conclusion, MANF was overexpressed in HCC and related to the occurrence and development of HCC. It is a potential diagnostic and prognostic indicator of HCC.

## 1. Introduction

Liver cancer is one of the most common human malignant gastrointestinal tumors and the fourth leading cause of cancer-related deaths worldwide [1, 2]. Hepatocellular carcinoma (HCC) characterized by its asymptomatic nature, high malignancy, early metastasis, and poor curative efficacy is responsible for >90% of primary liver cancers [3–5]. Despite recent therapeutic approaches such as surgical resection, radiofrequency ablation, and orthotropic liver transplantation, the prognosis of HCC remains poor. The metastasis and recurrence of HCC significantly reduce the survival rate and quality of life of HCC patients [5–8]. Therefore, novel biomarkers will be substantially beneficial for HCC diagnosis and treatment, and outcomes of HCC patients urgently need to be improved.

Mesencephalic astrocyte-derived neurotrophic factor (MANF), also named arginine-rich mutated in early tumors (ARMET), was first discovered as a new dopaminergic neurotrophic factor in astrocyte-conditioned medium by Petrova et al. in 2003 [9]. Apart from being secreted into the extracellular space, MANF has been found to remain inside the cells and localize in the endoplasmic reticulum (ER) lumen [10, 11]. Induction of ER stress *in vitro* causes upregulation of endogenous MANF expression [12, 13]. Hakonen et al. have shown that the protective effect of MANF is associated with inhibition of the nuclear factor- (NF-) *κ*B signaling pathway and alleviation of ER stress. MANF also enhances human beta cell proliferation when transforming growth factor- (TGF-) *β* signaling is inhibited [14]. In recent studies, ER stress has been shown to mediate HCC promoted by nonalcoholic fatty liver disease, and the NF-*κ*B pathway is closely associated with initiation of cancer [15, 16]. So the diagnostic value and clinical significance of MANF in HCC remain to be elucidated.

In this study, we investigated MANF expression in HCC cell lines, HCC tissues, and nontumor tissues by analyzing the data from bioinformation databases and confirmed our findings by Western blotting, polymerase chain reaction (PCR), and immunohistochemical staining. We examined the clinical and prognostic value of MANF in HCC patients.

## 2. Material and Methods

### 2.1. Ethics Statement

This study was approved by the Academic Committee of Shandong Provincial Hospital Affiliated to Shandong University and conducted according to the principles expressed in the Declaration of Helsinki. All the datasets were retrieved from the publishing literature, and all written informed consent was obtained. This article does not contain any studies with animals performed by any of the authors.

### 2.2. Patients and Specimens

A total of 311 patients undergoing hepatectomy between January 2011 and December 2014 were included in the study. HCC samples and paratumor tissues including 45 freshly frozen HCC samples, and 266 tissue microarrays (TMAs) were collected. We summarized their characteristics and study cohort diagram in Table S2. None of the patients had received chemotherapy or radiotherapy before we obtained the tissue specimens. The clinical staging was based on the 7th edition of the American Joint Committee on Cancer (AJCC) Staging System.

### 2.3. Real-Time PCR

Total RNA from liver tissues was isolated by the TRIzol reagent (Invitrogen, Life Technologies, Carlsbad, CA, USA), and 1 *μ*g mRNA was reverse transcribed to cDNA using the PrimeScript RT Reagent Kit Perfect Real Time (Takara Bio, Japan) according to the manufacturer's instructions. Reverse transcription- (RT-) PCR was conducted using the LightCycler 480 II Real-Time PCR System (Roche, Switzerland) with SYBR Green PCR Master Mix (Toyobo, Osaka, Japan). An initial denaturation at 95°C for 10 min was followed with PCR cycling: 94°C (30 s), 60°C (30 s), and 72°C (60 s) for 40 cycles. The primers of MANF were as follows: forward—5′-GTGCACGGACCGATTTGTAG-3′, reverse—5′-GGAAAGCTCCAGGCTTCACA-3′. The primers of *β*-actin were as follows: forward—5′-GAAGAGCTACGAGCTGCCTGA-3′, reverse—5′-CAGACAGCACTGTGTTGGCG-3′. Products were analyzed by melt curve analysis and agarose gel electrophoresis to determine product size and to confirm that no byproducts were formed. Results were expressed relative to the number of *β*-actin transcripts used as an internal control.

### 2.4. Western Blot Analysis

Liquid nitrogen frozen liver tissues were immersed in RIPA-added phenylmethylsulfonyl fluoride (100 : 1) (Beyotime, China) supplemented with protease and phosphatase inhibitors and sonicated on ice to obtain a homogenate. Specimens were centrifuged at 15 000 × *g* for 15 min, and the supernatant was used for Western blotting and ELISA. Concentration of the protein was assessed by BCA protein assay kit (Beyotime). Proteins were separated on SDS-PAGE and transferred to nitrocellulose membranes. After incubation with horseradish peroxidase-conjugated secondary antibodies for 2 h at room temperature, signals were detected by chemiluminescent reagents (Millipore, USA) and *β*-actin served as an internal control. The primary antibodies were as follows: rabbit anti-ARMET (Abcam, Cambridge, MA, USA; diluted 1 : 1000) and rabbit anti-*β*-actin (Cell Signaling Technology, Danvers, MA, USA; diluted 1 : 1000). Immunoreactivity was detected using the FluorChem Chemiluminescent Western Blot Imaging System (Cell Biosciences, Santa Clara, CA, USA).

### 2.5. Immunohistochemical (IHC) Detection of Tissue Microarray (TMA)

Two hundred and sixty-six HCC patients, including 259 who had follow-up information, were analyzed. For immunohistochemistry, 5 *μ*m tissue sections were prepared from each block. Tissue sections were deparaffinized, rehydrated, and rinsed in distilled water. After heating the sections in 10 mmol/L citrate buffer for antigen retrieval, the sections were incubated with primary antibody against ARMET (Abcam; dilution at 1 : 100) at 4°C, followed by secondary antibody for 1 h at room temperature. An intensity score of 0–3 was assigned for the intensity of tumor samples (0, none; 1, weak; 2, intermediate; and 3, strong) and the percentage of stained cells, assigning a score of 0–300. To assess the average degree of staining within a sample, multiple regions were analyzed. An *H* score was calculated using the following formula: *H* = (percentage of cells of weak intensity × 1) + (percentage of cells of moderate intensity × 2) + (percentage of cells of strong intensity × 3). The scoring was independently assessed by two assessors who were not aware of the clinical outcomes.

### 2.6. GEO Data Source

Meta-analysis of 24 sets of microarrays from the GEO database (http://www.ncbi.nlm.nih.gov/geo/) including 1475 HCC specimens and 981 nontumor specimens was used to evaluate the diagnostic power of MANF. The 24 cohorts consisted of GSE17548, GSE20140, GSE29722, GSE31370, GSE36411, GSE39791, GSE41804, GSE45050, GSE45267, GSE47595, GSE57958, GSE62232, GSE63898, GSE64041, GSE75285, GSE76311, GSE76427, GSE84006, GSE84402, GSE84598, GSE98383, GSE102083, GSE112791, and GSE121248 datasets. We summarized their characteristics such as cohort ID, RNA-seq platform, samples size (nontumor and tumor samples), publication year, and country in Table S1.

### 2.7. Statistics for Meta-analysis

Stata 12.0 was utilized to analyze the pooled diagnostic value of MANF with the data from the GEO dataset. *I*^2^ was used to evaluate the heterogeneity of those studies, which indicated significant heterogeneity at *I*^2^ > 50%. The random effects model was used, and subgroup analysis was performed to explore the source of heterogeneity, while heterogeneity was conspicuous between those studies. Publication bias was determined by Begg' s funnel plot and Egger's test.

### 2.8. ONCOMINE Analysis

ONCOMINE (http://www.oncomine.org/), an online cancer microarray database, was used to analyze differential expression classification in different cancers with their respective normal tissues and their clinical and pathological characteristics. MANF expression in HCC samples was compared with that in nontumor samples. The *P* value was generated utilizing Students' *t*-test. The cut-off *P* value and fold change were defined as 0.01 and 2, respectively.

### 2.9. GEPIA Dataset

The online database Gene Expression Profiling Interactive Analysis (GEPIA), providing customizable functions, is a newly developed interactive web server for analyzing the RNA sequencing expression data and prognostic value. Tumors and nontumor specimens in the GEPIA database were derived from The Genotype-Tissue Expression (GTEx) and The Cancer Genome Atlas (TCGA) projects (http://gepia.cancerpku.cn/index.html) [17]. Tumor/nontumor differential expression analysis, patient survival analysis, and correlation analysis were explored using the GEPIA database. We selected the median as the group cut-off for survival plots.

### 2.10. CCLE Dataset

Cancer Cell Line Encyclopedia (CCLE) project is a collaboration concentrated on a detailed genetic and pharmacological characterization of a large panel of human cancer cell lines, in order to develop integrated computational analyses that link distinct pharmacological vulnerabilities to genomic patterns and to translate cell line integrative genomics into clinical application. Genomic data, analysis, and visualization providing by CCLE for around 1000 cell lines are available for public access [18]. CCLE gene expression data of MANF were downloaded and collected from https://portals.broadinstitute.org/ccle/data.

### 2.11. LinkedOmics Dataset

LinkedOmics is a user-friendly bioinformatics web in the software ecosystem for disseminating data from large-scale cancer omics projects. It uses preprocessed and normalized data from the Broad TCGA Firehose and CPTAC data portal to reduce redundant efforts and focuses on exploration and interpretation of attribute associations and thus complements existing cancer data portals [19]. Correlation analysis data were collected and downloaded from http://www.linkedomics.org/admin.php.

### 2.12. EMBL-EBI Dataset

EMBL-EBI (https://www.ebi.ac.uk) is a user-friendly bioinformatics web and programmatic tool framework providing free and open access to a range of bioinformatics applications for sequence analysis [20]. The expression data of MANF in HCC cell lines was collected from the EMBL-EBI dataset.

### 2.13. Data Analysis and Statistics

SPSS version 22.0 (IBM Corporation, Armonk, NY, USA) and GraphPad Prism version 6.0 (GraphPad Software, La Jolla, CA, USA) were used for statistical analyses. We select the median expression level for splitting the high-expression and low-expression cohorts. Samples with expression level higher than this threshold are considered the high-expression cohort. Samples with expression level lower than this threshold are considered the low-expression cohort. The *χ*^2^ test was used to explore the correlation between MANF expression levels and the clinicopathological parameters. Survival analysis was performed by the Kaplan–Meier method. The relationship between different variables and survival was determined by the multivariate Cox proportional hazards method. The pooled diagnostic value of MANF in HCC was analyzed via receiver-operating characteristic (ROC) curves. The linear association between two variables was evaluated by Pearson's correlation. All of the data of samples are presented as the mean ± standard deviation (SD). The differences between tumor and nontumor samples were determined with nonparametric tests. In all cases, *P* < 0.05 was considered to be statistically significant.

## 3. Results

### 3.1. MANF Overexpression in HCC Was Explored by Analyzing Bioinformation Databases

**Figure 1:**
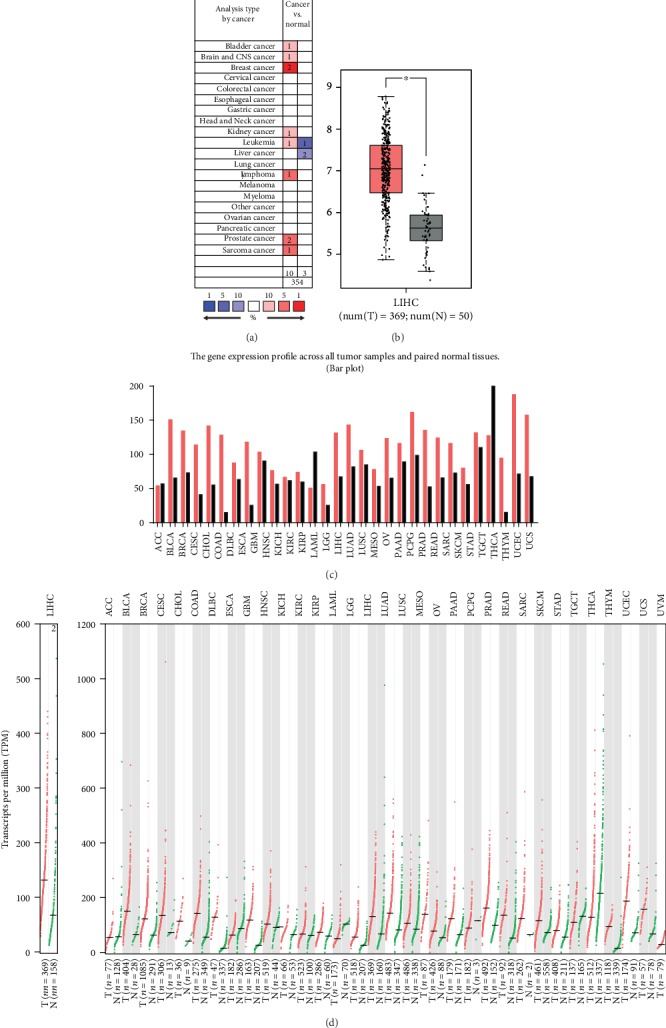
MANF expression levels in different types of human tumors. (a) Increased or decreased MANF in datasets of different tumors compared with normal tissues in the ONCOMINE database. Cell color is determined by the best gene rank percentile for the analyses within the cell. An analysis may be counted in more than one cancer type. (b) GEPIA generates box plots for comparing MANF expression in HCC (*n* = 369) and normal (*n* = 50) tissues (^∗^*P* < 0.01) (c) Bar plot of MANF expression profile across all tumor samples and paired normal tissues. (d) Human MANF expression levels in different tumor types from the TCGA database were analyzed by GEPIA. Bar height represents the median expression of certain tumor type or normal tissue.

We analyzed MANF mRNA expression in HCC tissues and paired nontumor tissues using the ONCOMINE and GEPIA databases. Compared to nontumor samples, ONCOMINE demonstrated that MANF was significantly upregulated in HCC samples (*P* < 0.01), while the other two statistics had no significance in this regard (Table 1). We compared transcriptional levels of MANF in cancer with those in normal tissues using ONCOMINE (Figure 1(a)). GEPIA showed that mRNA of MANF was significantly overexpressed in HCC samples and many other types of cancer (Figures 1(b)–1(d)).

**Figure 2:**
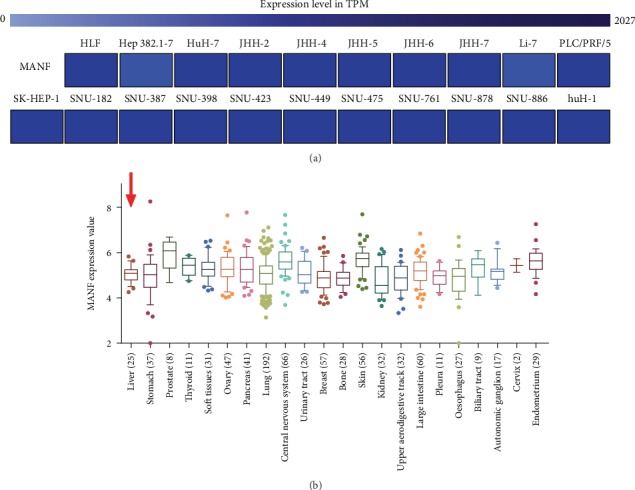
MANF expression levels in different types of cell lines. (a) Expression of MANF translational in HCC cell lines was tested by EMBL-EBI bioinformatics website. The darker the blue, the higher the level of MANF expression. (b) Expression of MANF in the cell lines was analyzed via CCLE databases. Liver cell lines are indicated by the red arrow.

We used the EMBL-EBI bioinformatics website to measure the expression of MANF in HCC cell lines, which indicated that MANF was upregulated in 21 HCC cell lines (Figure 2(a)). The CCLE database showed that MANF was highly expressed in a variety of cell lines originated from different tissue types (Figure 2(b)).

**Figure 3:**
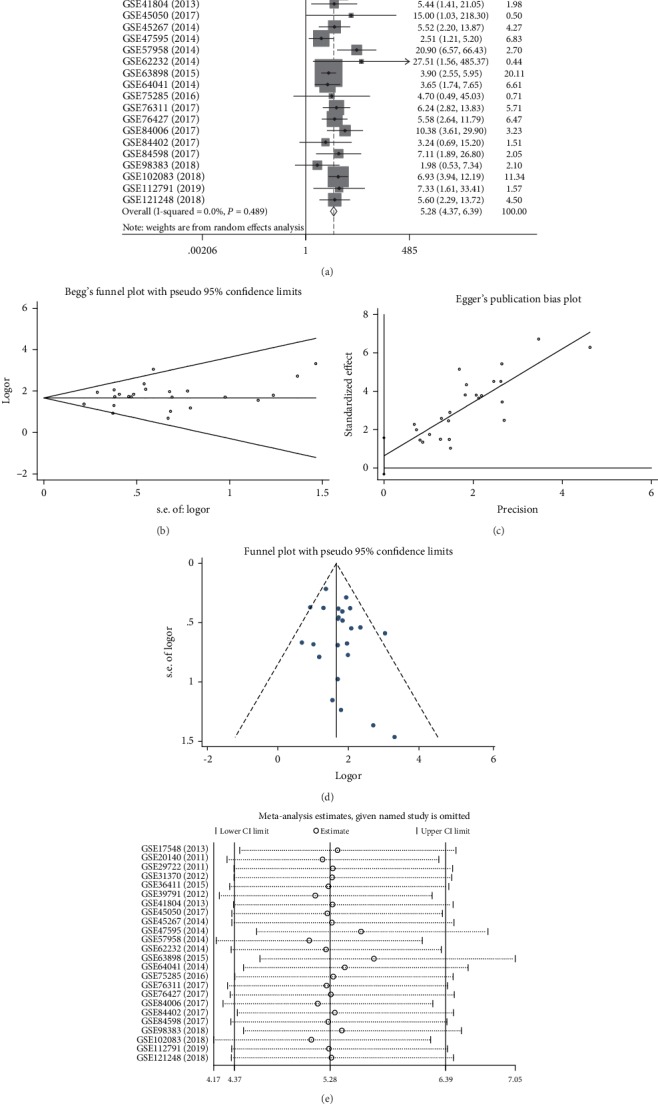
Meta-analysis for evaluating expression level of MANF in HCC. Each point represents a single microarray study. (a) Forest plot evaluating differences in MANF expression between HCC and nontumor tissues. Low and high MANF-expressing samples were regarded as the control and experimental groups, respectively. (b) Begg's test for the publication bias test of GEO databases. (c) Egger's test for the publication bias test of GEO databases. (d) Funnel plot for the publication bias test of GEO databases. (e) Sensitivity analysis was calculated by omitting each microarray in turn.

To explore further whether MANF expression was higher in HCC tissues than in nontumor tissues, 24 HCC microarrays from the GEO database were subjected to meta-analysis. Like the forest plot in Figure 3(a), higher MANF expression was found in HCC tissues than in the nontumor tissue [pooled odds ratio (OR) = 5.28, 95% confidence interval (CI) = 4.367–6.388, *I*^2^ = 0%, *P* = 0.489]. All the data were generated by a random effects model, and the *χ*^2^ test was used to analyze study heterogeneity. Publication bias was assessed with Begg's test, Egger's test, and funnel plots (Figures 3(b)–3(d)). There were no significant publication bias and heterogeneity. As shown in the sensitivity analysis, there were no significant differences between these microarrays (Figure 3(e)). Hence, high expression of MANF in HCC samples was identified by meta-analysis.

### 3.2. MANF Upregulated in HCC Was Confirmed by Experiments

**Figure 4:**
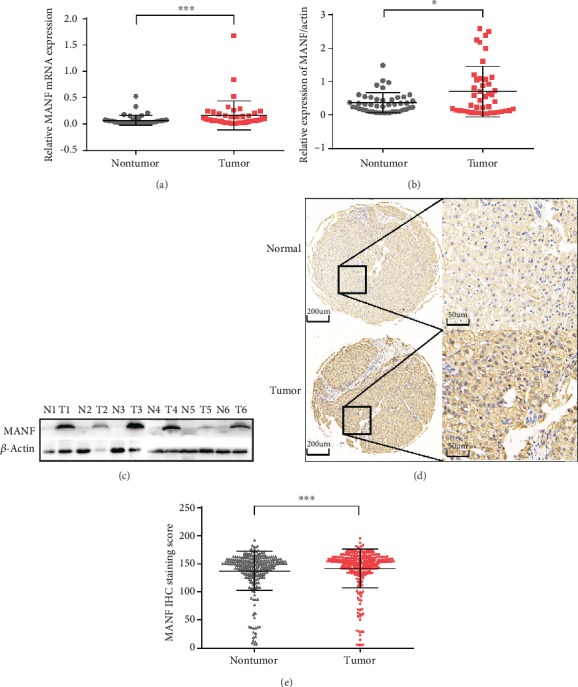
MANF expression levels in HCC compared with nontumor tissues. (a, b) MANF mRNA (^∗∗∗^*P* < 0.001) and protein (^∗^*P* < 0.05) expression levels in HCC clinical specimens compared with paired nontumor specimens were shown by box plots. (c) Representative Western blotting of HCC clinical specimens compared with paired nontumor specimens. N: normal tissue; T: tumor tissue. (d) Immunohistochemical analysis of MANF in HCC tissues and adjacent nontumor tissues previously analyzed by TMA (^∗∗∗^*P* < 0.001). (e) Representative MANF staining in HCC and normal tissues.

To confirm the expression level of MANF, we examined mRNA and protein levels of MANF in HCC and paired nontumor samples, utilizing quantitative RT-PCR and Western blotting. MANF expression in HCC tissues (*n* = 45) was higher than that in nontumor tissues (*n* = 45) (PCR, *P* < 0.05; Western blotting, *P* < 0.01) (Figures 4(a)–4(c)). We characterized MANF protein expression in human HCC and nontumor specimens by TMA. We analyzed MANF protein expression by immunohistochemical staining of HCC and paired nontumor tissues and found that MANF was significantly upregulated in HCC tissues compared with adjacent nontumor tissues (*n* = 266) (*P* < 0.01) (Figures 4(d) and 4(e)).

### 3.3. Diagnostic Value of MANF

**Figure 5:**
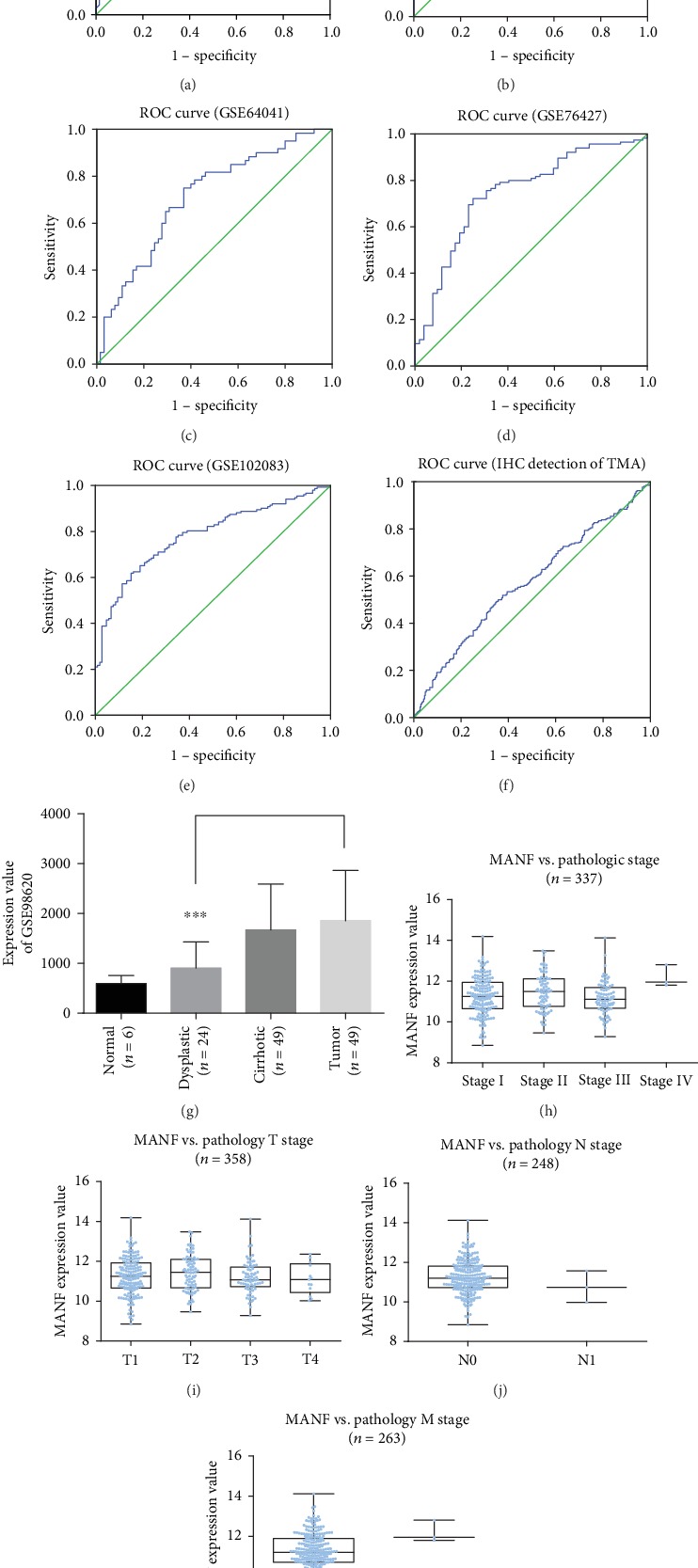
Diagnostic value of MANF in HCC was evaluated by ROC curve analysis among GEO databases. The blue line indicates HCC tissues while the green one indicates nontumor tissues. (a) ROC curve analysis of GSE39791 from GEO databases. (b) ROC curve analysis of GSE63898 from GEO databases. (c) ROC curve analysis of GSE64041 from GEO databases. (d) ROC curve analysis of GSE76427 from GEO databases. (e) ROC curve analysis of GSE102083 from GEO databases. (f) ROC curve analysis of TMA. (g) MANF expression value of different types of tissues in GSE98620 database (^∗∗∗^*P* < 0.001). (h–k) Correlation analysis of MANF expression and TNM staging obtained in LinkFinder of LinkedOmics.

The diagnostic value of MANF in identifying HCC and nontumor samples was evaluating by ROC curve analysis. Areas under the curve (AUCs) from GEO databases were as follows: GSE39791, 0.811 (95% CI: 0.740–0.882, *P* < 0.0001; Figure 5(a)) with cut-off point, and respective specificities and sensitivities were 10.075, 0.722, and 0.806; GSE63898, 0.677 (95% CI: 0.625–0.730, *P* < 0.0001; Figure 5(b)) with cut-off point, and respective specificities and sensitivities were 10.4102, 0.544, and 0.821; GSE64041, 0.710 (95% CI: 0.619–0.801, *P* < 0.0001; Figure 5(c)) with cut-off point, and respective specificities and sensitivities were 9.1892, 0.631, and 0.750; GSE76427, 0.749 (95% CI: 0.668–0.830, *P* < 0.0001; Figure 5(d)) with cut-off point, and respective specificities and sensitivities were 3357.82, 0.722, and 0.750; GSE102083, 0.782 (95% CI: 0.727–0.838, *P* < 0.0001; Figure 5(e)) with cut-off point, and respective specificities and sensitivities were 9.542, 0.618, and 0.848. AUCs from immunohistochemistry of TMA were 0.570 (95% CI: 0.522–0.619, *P* < 0.01; Figure 5(f)) with cut-off point, and respective specificities and sensitivities were 152.7620, 0.643, and 0.5. Results indicate that MANF was a reliable diagnostic marker in HCC.

To analyze expression levels of MANF in dysplastic nodules, the GEO database was searched. GSE98620 was the only database that meets the retrieval requirements. The result showed that higher MANF expression was found in HCC tissues than in dysplastic nodules (*P* < 0.001) (Figure 5(g)), and there was no statistical difference between normal tissues and dysplastic nodules.

Correlation analysis of MANF expression and TNM staging was performed using LinkFinder of LinkedOmics. There was no significant correlation between high MANF expression and TNM pathological stage (*P* > 0.05) (Figures 5(h)–5(k)).

### 3.4. Prognostic Value of MANF

**Figure 6:**
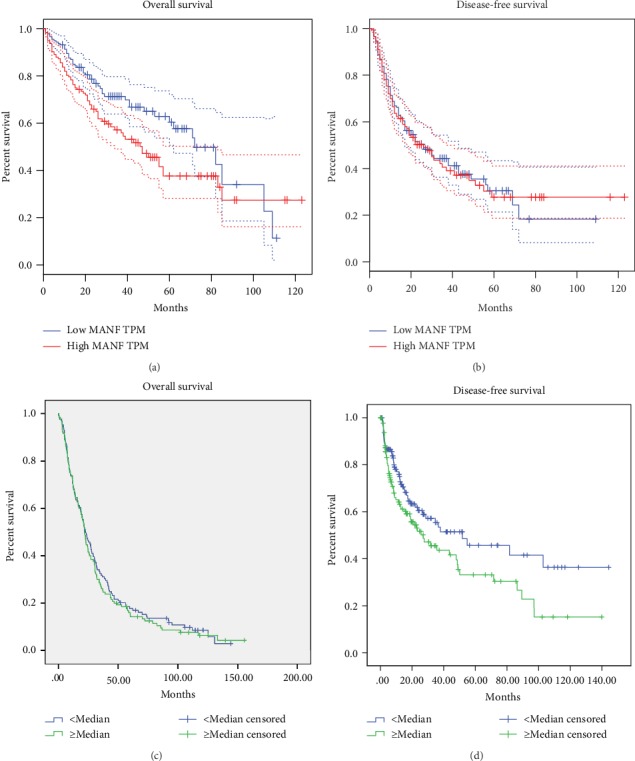
Correlation between MANF expression and clinical or prognostic parameters. (a, b) Association between MANF expression and overall survival and disease-free survival in HCC patients, from the GEPIA database. The log-rank test (Mantel–Cox test) was used to analyze the relationship between overall survival (*P* < 0.05), disease-free survival (*P* > 0.05), and MANF expression in patients with HCC. (c, d) Association between MANF expression and overall survival and disease-free survival in HCC patients from TMA analysis. The Kaplan–Meier test was used to analyze the relationship between overall survival (*P* > 0.05), disease-free survival (*P* < 0.05), and MANF expression in patients with HCC.

To investigate further the prognostic role of MANF in HCC patients, GEPIA database and supporting clinical data of TMA were analyzed. We analyzed TCGA prognostic data and MANF transcriptional level of HCC (*n* = 364) using the GEPIA database. The overall survival rates of HCC patients with high expression of MANF were significantly lower (*P* < 0.05) (Figure 6(a)) than those of patients with low expression of MANF. Disease-free survival did not differ significantly (Figure 6(b)). Beyond that, the TMA analysis of 259 HCC patients showed that patients with high MANF expression had shorter disease-free survival (*P* < 0.05) (Figure 6(d)) compared with patients with low expression of MANF. No significant difference was found in overall survival (Figure 6(c)). Therefore, high expression of MANF is a prognostic factor for HCC.

Patients with high MANF expression levels had a significantly higher risk of tumor recurrence (*P* < 0.05) (Table 2). There was no correlation of MANF expression with age, sex, *α*-fetoprotein (AFP) levels, hepatitis B virus infection, cirrhosis, tumor size, tumor number, TNM stage, differentiation grade, and venous invasion. Univariate Cox regression analysis showed that tumor number (*P* < 0.01), AFP level (*P* < 0.05), TNM stage (*P* < 0.05), and venous invasion (*P* < 0.001) were independent prognostic factors for HCC patients. Multivariate Cox regression analysis showed that only venous invasion (*P* < 0.001) was an independent prognostic factor for HCC (Table 3).

### 3.5. Coexpression Genes Correlated with MANF in HCC

**Figure 7:**
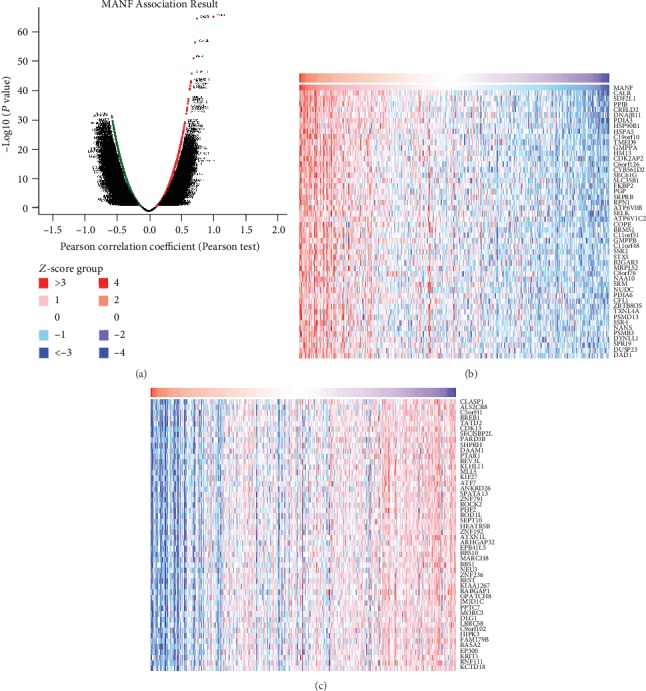
Genes differentially expressed in correlation with MANF in HCC (LinkedOmics). (a) Correlations between MANF and genes differentially expressed in HCC were analyzed by Pearson's test. (b, c) Genes positively and negatively correlated with MANF in HCC are shown by heat maps (TOP 50). Red dots indicate positively correlated genes, and green and blue dots indicate negatively correlated genes.

MANF association results were confirmed using LinkFinder of LinkedOmics to analyze mRNA sequencing data from 367 HCC patients in the TCGA via Pearson's correlation test. The volcano plot (Figure 7(a)) shows that there were 3773 genes positively correlated with MANF (marked by red dots) and 4404 genes negatively correlated (marked by green dots) (*P* < 0.01, FDR < 0.01). The top 50 significant gene sets positively and negatively correlated with MANF are shown in the heat map (Figures 7(b) and 7(c)). As it turns out, MANF has extensive influence on the transcriptome.

**Figure 8:**
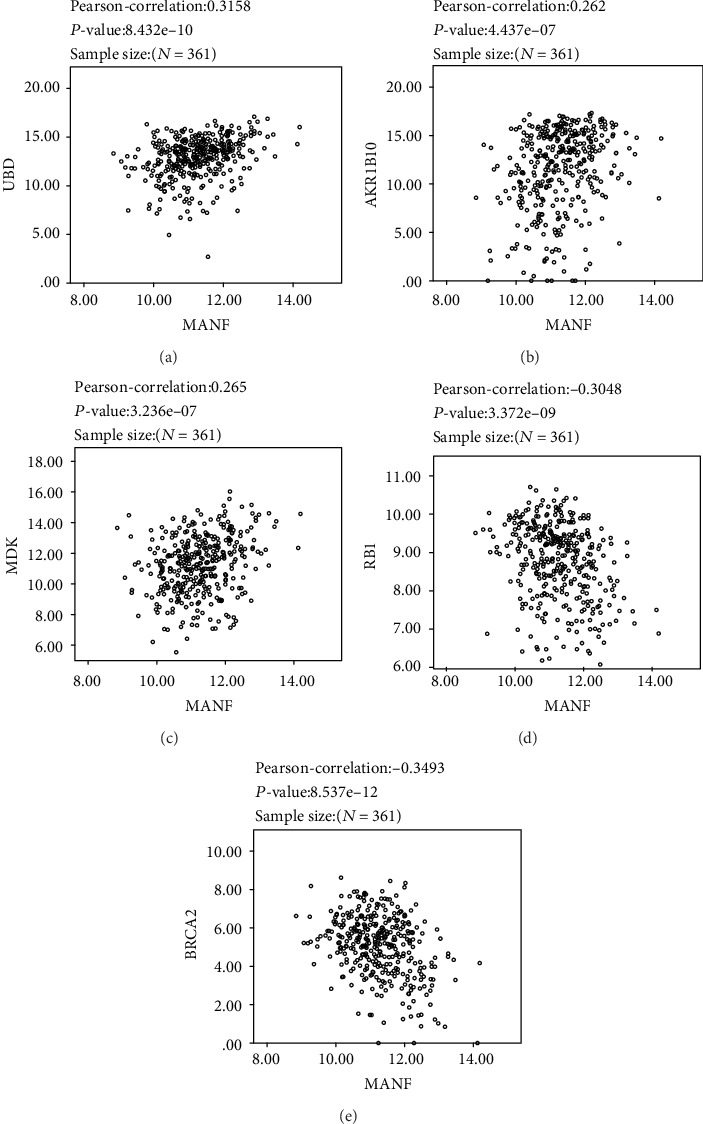
Genes correlated with MANF in HCC. (a–c) Correlations between MANF and the top 10 genes including *UBD*, *MDK*, and *AKR1B10*, which have the highest expression multiples in HCC (*P* < 0.01). (d, e) Correlations between MANF and tumor suppressor genes *RB1* and *BRCA2* (*P* < 0.01).

We examined the correlations between MANF and the top 10 genes with the highest expression multiples in HCC. MANF was significantly correlated with *UBD*, *MDK*, and *AKR1B10* and had some degree of correlation with other genes (Figures 8(a)–8(c)).

To confirm the role of MANF expression in the development of cancer, we used LinkFinder or LinkedOmics to analyze the relationship with common oncogenes and tumor suppressor genes. There were negative correlations between MANF expression and *RB1* (Pearson's correlation = ‐0.3048, *P* < 0.01) and *BRCA2* (Pearson's correlation = ‐0.3493, *P* < 0.01) (Figures 8(d) and 8(e)).

## 4. Discussion

HCC accounts for >90% of the histological types of primary malignant liver tumors, which are highly malignant and have a high recurrence rate and poor prognosis [3, 4]. Therefore, elucidating the molecular mechanisms underlying the progression and initiation of HCC is important for treatment selection.

ER stress can be induced by oncogene activation, such as *B-Raf* proto-oncogene mutations, *H-Ras* proto-oncogene mutations, and *c-Myc* amplification, as well as chemotherapeutic drugs [21]. When the ER functions, only correctly folded proteins can reach their cell compartment and unfolded or misfolded proteins accumulate within the ER lumen. Overwhelming cellular demand and shortage of cellular energy availability lead to the accumulation of wrongly folded proteins [22]. Unfolded protein response (UPR) helps cells to reestablish homeostasis by decreasing protein synthesis and increasing the folding and clearance capacity of the ER [23]. Under sustained ER stress conditions, ER homeostasis mediated by UPR cannot be restored and leads to initiation of apoptosis [24]. However, cancer cells have evolved UPR to alleviate ER stress conditions as a survival mechanism for progression [25, 26]. MANF protects SH-SY5Y cells against 6-OHDA-induced toxicity by activating the PI3K/Akt/mTOR pathway and alleviating ER stress [27]. ER stress regulated by UPR also plays an important role in mechanisms of chemotherapy or radiation resistance in cancer [28]. MANF is a neurotrophic factor secreted from cells [29]. Kim et al. have indicated that MANF can serve as a urinary biomarker for detecting ER stress in podocytes or renal tubular cells [30]. Expression of MANF has been confirmed to be closely related to ER stress, which is a mediator in the initiation of HCC [16].

The liver is an important organ for the synthesis of proteins and lipids, so hepatocyte ER has appropriate adaptive capacity [31]. When the liver is in a state of inflammation for a long time, ER stress is maintained at a high level, which leads to hepatic dysfunction and progression of liver diseases, even HCC [32].

Our study is believed to be the first to explore mRNA expression and prognostic value of MANF in HCC. We analyzed MANF expression in HCC samples using gene expression and clinical prognostic data in the TCGA, CCLE, EMBL-EBI, GEPIA, LinkedOmics, and ONCOMINE databases, clinical specimens from our hospital, and HCC TMAs. We found that MANF was always highly expressed in HCC and many other cancers, indicating the significance of MANF in tumorigenesis.

Although previous studies have shown that MANF is highly expressed in HCC, there is a lack of reliable means to prove the diagnostic value of MANF in HCC. Therefore, we conducted a meta-analysis of MANF expression in previous studies retrieved from the GEO HCC dataset. ROC curves from GEO datasets were used to confirm the satisfactory diagnostic performance of MANF. However, diagnostic performance of MANF in TMA analysis was not entirely satisfactory, which may be caused by the subjectivity of immunohistochemical staining analysis. Overall, MANF was shown to be a potential diagnostic marker for distinguishing between HCC and nontumor tissues.

As shown in the analysis of the GEPIA database and TMA supporting clinical data, MANF is a novel potential prognostic marker for HCC patients. Consistent with these findings, patients with high MANF expression levels had a higher risk of tumor recurrence. Dysfunction of ER stress and UPR signal underline the resistance of cancer cells to chemotherapy, and ER stress response was inhibited in chemoradiotherapy-resistant cells compared with that in sensitive cells [33]. MANF could alleviate ER stress and reduce ER stress-induced cell death, and ER stress activation could cause upregulation of MANF in vivo and in vitro [13, 34]. The higher recurrence rate and worse prognosis might be due to MANF-ER stress-mediated chemotherapy or targeted drug resistance; it needs further validation.

Our study proved that MANF was upregulated in HCC tissues more than in nontumor tissues. High expression of MANF was also involved in the development and progression of HCC and a potential indicator in the diagnosis, treatment, and prognosis of HCC. In addition, the molecular mechanism involved in MANF expression and occurrence of HCC remains unknown. In order to study further the important role of MANF in occurrence and development of HCC, more *in vitro* and *in vivo* experiments should be conducted.

## 5. Conclusion

MANF was overexpressed in HCC and related to poor prognosis and progression of HCC. Our results showed that MANF is a potential diagnostic and prognostic indicator of HCC.

## Figures and Tables

**Table 1:** The significant changes of MANF expression in transcription level of HCC vs. normal tissues (ONCOMINE database).

Tissue types	Fold change	*P* value	*t*-test	References	Overexpression gene rank
HCC vs. normal	1.945	1.54*E*-53^∗∗^	1.77*E*+01	Roessler Liver 2 Statistics (Roessler et al., Cancer Res 2010/12/15)	386 of 12,624 measured genes (in top 4%)
HCC vs. normal	1.398	0.0000514^∗∗^	3.984	Chen Liver Statistics (Chen et al., Cancer Res 2010/12/15)	1850 of 10,802 measured genes (in top 18%)
HCC vs. normal	1.435	0.011^∗^	2.391	Roessler Liver Statistics (Roessler et al., Cancer Res 2010/12/15)	3333 of 12603 measured genes (in top 27%)
HCC vs. normal	1.482	0.122	1.236	Wurmbach Liver Statistics (Wurmbach et al., Hepatology 2007/04/01)	7466 of 19,574 measured genes (in top 39%)
HCC vs. normal	-2.279	1	-6.273	Mas Liver Statistics (Mas et al., Mol Med 2008/12/21)	12189 of 12,603 measured genes (in top 97%)

Notes: ^∗^*P* < 0.05; ^∗∗^*P* < 0.01.

**Table 2:** The relationship between MANF status and clinicopathological features of HCC (tissue microarray).

Clinicopathological features	Number of cases (*n*)	MANF expression, *n* (%)		*P* value
		High	Low	
Age				
≥Median	133	64 (48.1)	69 (51.9)	0.54
<Median	133	69 (51.9)	64 (48.1)	
Gender				
Male	241	123 (51)	118 (49)	0.293
Female	25	10 (40)	15 (60)	
HBV				
Positive	245	124 (50.6)	121 (49.4)	0.495
Negative	21	9 (42.9)	12 (57.1)	
Cirrhosis				
Positive	220	108 (49.1)	112 (50.9)	0.517
Negative	46	25 (54.3)	21 (45.7)	
Tumor size				
≥5	221	106 (48)	115 (52)	0.141
<5	45	27 (60)	18 (40)	
Tumor number				
Single	153	73 (47.7)	80 (52.3)	0.385
Multiple	113	60 (53.1)	53 (46.9)	
AFP				
≥20	201	95 (47.3)	106 (52.7)	0.117
<20	65	38 (58.5)	27 (41.5)	
TNM stage				
Stage I-II	124	68 (54.8)	56 (45.2)	0.104
Stage III-IV	142	65 (45.8)	77 (54.2)	
Differentiation grade				
Grade 1-2	182	94 (51.6)	88 (48.4)	0.429
Grade 3-4	84	39 (46.4)	45 (53.6)	
Vasoinvasion				
Yes	53	28 (52.8)	25 (47.2)	0.645
No	213	105 (49.3)	108 (50.7)	
Tumor recurrence				
Yes	110	64 (58.2)	46 (41.8)	0.025 ^∗^
No	156	69 (44.2)	87 (55.8)	

Notes: ^∗^*P* < 0.05; ^∗∗^*P* < 0.01.

**Table 3:** Univariate and multivariate analyses of prognostic variables for overall survival in HCC patients.

Clinicopathological features	Univariate analysis			Multivariate analysis		
	HR	95% (CI)	*P* value	HR	95% (CI)	*P* value
MANF expression (T)						
Low	1.000					
High	1.070	0.829-1.380	0.605			
MANF expression (NT)						
Low	1.000					
High	0.896	0.694-1.158	0.401			
Age						
<Median	1.000					
≥Median	1.014	0.786-1.307	0.917			
Gender						
Male	1.000					
Female	0.751	0.470-1.202	0.233			
HBV						
Negative	1.000					
Positive	1.169	0.731-1.870	0.514			
Cirrhosis						
Negative	1.000					
Positive	0.931	0.664-1.304	0.677			
Tumor size						
<5	1.000					
≥5	1.218	0.869-1.705	0.252			
Tumor number						
Single	1.000			1.000		
Multiple	1.442	1.114-1.867	0.005^∗∗^	1.278	0.955-1.710	0.099
AFP						
<20	1.000			1.000		
≥20	1.360	1.013-1.827	0.041^∗^	1.142	0.838-1.555	0.401
TNM stage						
Stage I-II	1.000			1.000		
Stage III-IV	1.404	1.086-1.814	0.010^∗^	0.991	0.726-1.353	0.957
Differentiation grade						
Grade 1-2	1.000					
Grade 3-4	0.955	0.725-1.260	0.746			
Vasoinvasion						
No	1.000			1.000		
Yes	2.757	1.993-3.813	<0.001^∗∗^	2.521	1.769-3.591	<0.001^∗∗^
Tumor recurrence						
No	1.000					
Yes	0.815	0.630-1.054	0.119			

Notes: ^∗^*P* < 0.05; ^∗∗^*P* < 0.01. T: tumor tissue; NT: nontumor tissue.

## Data Availability

The PCR, WB, immunohistochemical staining and their supporting clinical data used to support the findings of this study are available from the corresponding author upon request because the data also forms part of an ongoing study. The Microarray Data supporting this META-ANALYSIS are from previously reported studies and datasets, which have been cited. The processed data are available at Gene Expression Omnibus (GEO) database (http://http://www.ncbi.nlm.nih.gov/geo/). The bioinformation databases data supporting this study are from previously reported studies and datasets, which have been cited. The processed data are available at ONCOMINE (http://www.oncom/http://ine.org/), Gene Expression Profiling Interactive Analysis (GEPIA) (http://gepi.a.cancer-pku.cn/), Cancer Cell Line Encyclopedia (CCLE) (https://portals.brohttp://adinstitute.org/ccle/data), LinkedOmics (http://www.linkedomics.org/admin.php), EMBL-EBI (https://www.ebi.ac.uk).

## Abbreviations

HCC: Hepatocellular carcinoma
MANF: Mesencephalic astrocyte-derived neurotrophic factor
(ARMET): Arginine-rich mutated in early tumor
GEPIA: Gene Expression Profiling Interactive Analysis
NAFLD: Nonalcoholic fatty liver disease
TCGA: The Cancer Genome Atlas
EMBL-EBI: The European Bioinformatics Institute
CCLE: Cancer Cell Line Encyclopedia
GEO: Gene Expression Omnibus
SMD: Standard mean difference
TMA: Tissue microarray
ROC: Receiver-operating characteristic.

## Supplementary Materials

Supplementary Materials Supplementary material is the basic characteristics of 24 HCC cohort from GEO supporting meta-analysis in this study.
