# Supplementary material for: Diagnostic and Prognostic Values of MANF Expression in Hepatocellular Carcinoma
Source: Biomed Res Int. 2020 Apr 22;2020:1936385. doi: 10.1155/2020/1936385 (PMC7193290; doi:10.1155/2020/1936385)
Supplement: Supplementary Materials — Supplementary material is the basic characteristics of 24 HCC cohort from GEO supporting meta-analysis in this study. [file 1936385.f1.docx]

**Table S1:** Basic characteristics of 24 HCC cohort from GEO

| **Cohort ID** | **Publication**  **year** | **Region** | **Platform** | **Number of samples** | |
| --- | --- | --- | --- | --- | --- |
|  |  |  |  | **Nontumor** | **Tumor** |
| GSE17548 | 2013 | non-Asia | GPL570 | 20 | 17 |
| GSE20140 | 2011 | non-Asia | Other | 34 | 35 |
| GSE29722 | 2011 | non-Asia | GPL570 | 10 | 10 |
| GSE31370 | 2012 | Asia | GPL10558 | 5 | 15 |
| GSE36411 | 2015 | Asia | GPL10558 | 42 | 42 |
| GSE39791 | 2012 | non-Asia | GPL10558 | 72 | 72 |
| GSE41804 | 2013 | Asia | GPL570 | 20 | 20 |
| GSE45050 | 2017 | non-Asia | GPL6244 | 8 | 6 |
| GSE45267 | 2014 | Asia | GPL570 | 41 | 46 |
| GSE47595 | 2014 | non-Asia | Other | 48 | 88 |
| GSE57958 | 2014 | Asia | GPL10558 | 39 | 39 |
| GSE62232 | 2014 | non-Asia | GPL570 | 10 | 81 |
| GSE63898 | 2015 | non-Asia | Other | 168 | 228 |
| GSE64041 | 2014 | non-Asia | GPL6244 | 65 | 60 |
| GSE75285 | 2016 | non-Asia | GPL570 | 5 | 50 |
| GSE76311 | 2017 | non-Asia | Other | 58 | 61 |
| GSE76427 | 2017 | Asia | GPL10558 | 52 | 115 |
| GSE84006 | 2017 | Asia | Other | 38 | 38 |
| GSE84402 | 2017 | Asia | GPL570 | 14 | 14 |
| GSE84598 | 2017 | non-Asia | GPL10558 | 22 | 22 |
| GSE98383 | 2018 | non-Asia | GPL570 | 53 | 11 |
| GSE102083 | 2018 | Asia | GPL570 | 105 | 152 |
| GSE112791 | 2019 | Asia | GPL570 | 15 | 183 |
| GSE121248 | 2018 | Asia | GPL570 | 37 | 70 |

**Table S2:** Patient characteristics and study cohort diagram.

| **Clinicopathological features** | **Number of cases** | **Number of cases in** | **Total number of** |
| --- | --- | --- | --- |
|  | **in 266 TMA** | **45 frozen samples** | **311 cases** |
| **Age** |  |  |  |
| ≥60 | 56 | 13 | 69 |
| <60 | 210 | 32 | 242 |
| **Gender** |  |  |  |
| male | 241 | 36 | 277 |
| Female | 25 | 9 | 34 |
| **HBV** |  |  |  |
| Positive | 245 | 36 | 281 |
| Negative | 21 | 9 | 30 |
| **Cirrhosis** |  |  |  |
| Positive | 220 | 38 | 258 |
| Negative | 46 | 7 | 53 |
| **Tumor size** |  |  |  |
| ≥5 | 221 | 29 | 250 |
| <5 | 45 | 16 | 61 |
| **Tumor number** |  |  |  |
| Single | 153 | 34 | 187 |
| Multiple | 113 | 11 | 124 |
| **AFP** |  |  |  |
| ≥20 | 201 | 25 | 226 |
| <20 | 65 | 20 | 85 |
| **TNM stage** |  |  |  |
| Stage Ⅰ-Ⅱ | 124 | 27 | 151 |
| Stage III-IV | 142 | 18 | 160 |
| **Differentiation grade** |  |  |  |
| Grade 1-2 | 182 | 26 | 208 |
| Grade 3-4 | 84 | 19 | 103 |
| **Vasoinvasion** |  |  |  |
| YES | 53 | 4 | 57 |
| NO | 213 | 41 | 254 |
| **Tumor recurrence** |  |  |  |
| YES | 110 |  | 110 |
| NO | 156 |  | 156 |

**Note:** All the patients were Asian
